# Investigating the association between the symptoms of women with Fibromyalgia, Digestive function, and markers of the microbiota of the Gastrointestinal Tract (The FIDGIT Study): study protocol

**DOI:** 10.1186/s12891-023-06259-3

**Published:** 2023-02-27

**Authors:** Sharon Erdrich, Jason A Hawrelak, Stephen P Myers, Momchilo Vuyisich, Joanna E Harnett

**Affiliations:** 1 Faculty of Medicine and Health, School of Pharmacy, The University of Sydney, Sydney, New South Wales Australia; 2 College of Health and Medicine, University of Tasmania, Hobart, Tasmania Australia; 3 National Centre for Naturopathic Medicine, Faculty of Health, Southern Cross University, Lismore, New South Wales Australia; 4 NatMed Research, Evans Head, New South Wales, Australia; 5 Viome Life Sciences Inc, Seattle, Washington, United States of America

**Keywords:** Fibromyalgia, Microbiome, Functional Gastrointestinal Disorders, Study Protocol

## Abstract

**Background:**

Fibromyalgia a common idiopathic condition affecting around 1.4% of adults globally. Its signature symptom is chronic widespread pain, with a constellation of somatic and psychological symptoms. Fibromyalgia is associated with significant reductions in quality of life, yet to date there is no biochemical marker for its diagnosis. Previous studies have indicated a strong association with gastrointestinal dysfunction, and more recently, alterations to the gut microbiome. No studies have examined the inter-relationship between fibromyalgia, gastrointestinal dysfunction, and the microbiome. This prospective observational case-controlled study will gather data on gastrointestinal function, dietary intake, fermentation patterns of ingested carbohydrates, and symptoms commonly associated with fibromyalgia. These will be evaluated alongside human gene expression and metatranscriptomic analysis of the oral and faecal microbiome.

**Methods:**

Adult women aged ≥18 years diagnosed with fibromyalgia and/or meeting ACR 2016 criteria, and healthy family or age-matched controls will be recruited from the community. From consenting participants, we will collect detailed survey information and samples of blood, urine, stool, saliva, and breath.

**Discussion:**

This is the first prospective study examining interactions between digestive function, human gene expression, and the gut microbiome together with general, and fibromyalgia-specific, symptoms experienced by New Zealand women. This exploration will allow an in-depth understanding of clinically relevant factors that are associated with fibromyalgia and will guide further research and contribute to improved management of this poorly understood condition.

**Trial Registration:**

The study was approved by the New Zealand Health and Disability Committee (HDEC) (ref: 20/CEN/197) and registered with the Australia and New Zealand Clinical Trials Registry (ANZCTR), registration number ACTRN12620001337965. Written consent will be obtained after providing participants with detailed information about the procedures. Access to data will be restricted to the immediate research team, and all samples and survey data will be deidentified and coded before analysis.

## Background

Fibromyalgia is a common, chronic rheumatological condition of unknown aetiology. Since it was first described in 1592 as muscular rheumatism, its definition has evolved [1], and fibromyalgia is now considered a syndrome of widespread body pain, associated with a constellation of symptoms, such as fatigue, depression, headaches, waking unrefreshed and abdominal pain or cramps [2].

The prevalence of fibromyalgia (also previously known as myofascial pain syndrome, fibrositis and fibromyositis) [1] ranges from 0.2% to 6.6%, with women more commonly afflicted than men [3]. It is associated with substantive decreases in quality of life and significant socioeconomic burden, with high direct and indirect costs [4].

The pathophysiology of fibromyalgia remains elusive, which poses challenges for diagnosis and treatment. While there is no clear inflammatory process, several candidates have been proposed as predisposing or exacerbating factors, including infection, oxidative stress, pre-existing autoimmune conditions, genetics, sleep disorders, nervous system dysfunction, changes in epidermal nerve fibres, and hormonal dysregulation [5]. While none have proven to be universal or predictive of the condition, recent guidelines recommend focusing on immunological markers, specifically auto-immune antibodies [6].

There are currently no laboratory evaluations that aid accurate determination of fibromyalgia. Testing is done to confirm absence of other pathology, leading to a diagnosis based on subjective evaluation of clinical presentation of pain and concomitant symptoms [2]. Management of fibromyalgia typically centres around mitigation of the predominant symptoms (pain, sleep, and mood) with pharmaceutical agents and supportive therapies such as stress management and exercise commonly recommended [7].

Among the range of associated somatic symptoms is a high prevalence of gastrointestinal dysfunction. Functional gastrointestinal disorders (FGIDs), recently redefined as *Disorders of Gut-Brain Interaction (DGBI)* [8], encompass a range of gastrointestinal disorders for which no structural basis can be found. Symptoms are believed to be due to interactions between the gut microbiota, immune function in the gastrointestinal tract, altered sensitivity of the viscera, and dysregulation of the central nervous system. Irritable bowel syndrome (IBS) is the most common of the FGIDs, and is commonly comorbid in people with fibromyalgia, as explored in our systematic review [9]. Additional reports have demonstrated that impaired digestive dysfunction impacts the severity of fibromyalgia symptoms [10, 11]. The FGIDs are increasingly associated with alterations in the composition, diversity, and function of the gastrointestinal microbiota [12], with recent findings of distinctive differences between the gut microbiome in those with fibromyalgia compared to healthy controls [13]. Existing literature exploring relationships associated with the gut microbiota and its biochemical markers were explored in an additional systematic review [14].

The concept of a gut-musculoskeletal axis is a recent one. Several targets for interaction have been proposed, including gut microbial interactions with available nutrients [15], impaired intestinal integrity [16], modulation of immune cell function [17], and translocation of microbial by-products, such as lipopolysaccharides (LPS) from gram-negative organisms. Notably, recent explorations into associations between both composition and function of gut microbiota and fibromyalgia, discovering demonstrable links. Minerbi et al [18] identified gut-microbiota associated serum markers that were able to differentiate between fibromyalgics and controls. Work is ongoing in this area.

Additionally, a gut-microbiome-immune axis has also been proposed [19]. Mechanisms subsequent to impaired intestinal barrier have been described as important predisposing, or possibly instrumental, factors in the development of a range of autoimmune diseases [16, 20]. Microbial LPS is associated with immunosuppression, and an inability to tolerate this endotoxin is a candidate for contribution to development of autoimmunity [21]. A range of additional proposed mechanisms are explored by Li et al [22]. Yet to be confirmed is cause versus effect in these relationships, and whether fibromyalgia is an autoimmune condition or not.

To date, no studies have explored the intersection between the gut (oral and faecal) microbiota, the FGID/DGBI and the complex symptom presentation in people with fibromyalgia. The FIDGIT study explores this intersection and aims to provide insights into these relationships.

## Objectives

The primary aim of this clinical study is to investigate the relationships between the composition and function of the oral and gastrointestinal microbiota, digestive symptoms, and clinical presentation of adult women living with fibromyalgia and compare these to age or family-matched healthy female controls.

The specific aims are to assess and contrast: (A) the interaction between the oral and faecal microbiome, including its function, complexity, and diversity, and (i) digestive symptoms; (ii) symptoms associated with fibromyalgia; (iii) human gene expression, and (B) the interaction between digestive symptoms and symptoms associated with fibromyalgia. We will also examine these factors in relationship to the human transcriptome.

Factors known to influence the composition of the gut microbiome, including oral health, diet, sleep, body composition, medication and supplement use will be evaluated. We will also evaluate redox balance, glycaemic control, and environmental mould exposure.

The study is designed to test the hypothesis that the gut microbiome, and its transcriptome, is associated with digestive function, and the symptoms associated with fibromyalgia.

Here we detail the study protocol, recruitment strategy and analytical procedures.

## Methods/study design

### Conceptual framework and study design

We will investigate associations between the oral and faecal metatranscriptome, human transcriptome, FGIDs, indices of intestinal carbohydrate fermentation, diet, oxidative stress, and symptoms associated with fibromyalgia.

### Participants and recruitment

This is a prospective, single-centre, cross-sectional case-controlled, observational study. We aim to recruit at least 100 adult women meeting fibromyalgia diagnostic criteria and a minimum of 50 matched controls. Limiting the study to females reduces the confounding influence of gender difference on gastrointestinal function and the gut microbiome. To determine suitability for inclusion as a participant or as a healthy control, initial screening will be conducted via a survey hosted on Research Electronic Data Capture (REDCap) (via the University of Sydney). Prospective volunteers will then be contacted directly by the study co-ordinator to confirm suitability and book appointments as appropriate.

#### Sample size

Based on 2002 data, the estimated prevalence of fibromyalgia in the NZ population (~5 million) is 1.5% [23]. Up to 98% of women with fibromyalgia may have at least one FGID [24], thus for alpha of 0.05 and confidence interval (CI) of 95%, no less than 55 participants and 55 controls are required.

#### Inclusion and exclusion criteria

Women aged 18 to 75 years, meeting fibromyalgia criteria as defined by the American College of Rheumatology (2016) (ACR2016), or with a formal diagnosis by a medical practitioner, and willing to comply with study requirements, including travelling to the study site in Auckland, NZ, will be invited to join the study.

Exclusion criteria for both groups include being a current smoker, having a diagnosis of diabetes mellitus, being pregnant or breastfeeding, having any major comorbid illness (e.g., malignancy, active inflammatory or metabolic disease), or being unable to withhold anti-inflammatory medication for at least 48 hours. At least 4 weeks must lapse after any antibiotic treatment before enrolment.

Healthy controls must meet the same criteria, except they must *not* meet criteria for fibromyalgia.

#### Recruitment

A study website has been designed to enhance the recruitment phase of this study. The site contains information about the project, details on the study design, and a link to the eligibility survey, hosted on REDCap. Links to this website, along with summary information, will be provided to a range of health professionals thought to have interactions with people with fibromyalgia. Printed flyers will be distributed to medical centres, allied health clinics and pharmacies, and displayed on community and library noticeboards within a 5km radius of the study site. As each participant completes submission of study samples, a report detailing the available results will be sent to them and their medical practitioner. A brief outline of the study will be included, along with a recruitment flyer.

In addition, the study will be promoted via advertisements and social media posts via support groups and community pages, including the Facebook page of Fibromyalgia NZ.

Volunteers with fibromyalgia will be requested to invite a healthy adult female who is a family member (sister, daughter, mother), household cohabitant or close friend to participate to form our control group. Where numbers for controls are lacking, age-matched controls will be sought via social and professional networks.

In lieu of financial compensation to volunteers, each participant will receive a report detailing results from tests analysed at the study site. Together these have a retail value of more than NZ$500.

## Study procedures

The FIDGIT Study will be conducted at House of Health in Auckland, NZ, with recruitment commencing November 2021, under supervision of the University of Sydney. All eligible volunteers will be required to attend the study site on at least one, and on up to 4 occasions.

At the initial visit, after satisfying any questions and obtaining informed consent, a unique participant identifier will be allocated. A trained technician will perform baseline assessments and phlebotomy. Details of current medication and supplement use will be documented, and procedures for at-home sample collection outlined and equipment provided. To augment the verbal and written information, some at-home tests will have additional animated instructions published on YouTube (see Declarations).

At the first visit, participants will proceed to a hydrogen-methane breath test, as detailed under *Breath Testing*. While undertaking breath testing, the comprehensive integrated questionnaire and the dietary assessment tool are completed.

## Clinical measurements

All volunteers will attend the enrolment appointment after fasting for a minimum of 12 hours (water only), having withheld dietary supplements and anti-inflammatory medication for at least 48 hours, and before taking any morning medications. After screening for risk of recent exposure to, or symptoms of, SARS-CoV2 infection, including heart rate, peripheral oxygen saturation, and temperature, volunteers will be weighed without shoes on an electronic scale and documented to the nearest 0.1kg. Height will be measured to the nearest 0.5 cm using a wall-mounted stadiometer. These data are used to calculate body mass index (BMI) using the formula: body weight (kg)/height^2^ (m). Systolic and diastolic blood pressure will be measured (while seated) by the study co-ordinator (a registered nurse) with a manual sphygmomanometer on the arm not used for venepuncture.

## Medication and dietary supplements

Prospective participants are asked to list their current medications and dietary supplements when completing the eligibility survey. This will aid identification of conditions that trigger an exclusion flag and aid the study co-ordinator’s instructions to participants regarding medication and the required fasting and exclusion period. The list of medications and supplements will be verified at the initial appointment, with determination of duration of use. These are then catalogued in an Excel® spreadsheet; medications according to the ATC/DDD drug classification system [25], to the second level, or the third level for gastrointestinal system-specific drugs. Supplements will be coded according to the Australian Health Survey (AHS) dietary supplement 5-digit classification code [26].

## Sample collection & analysis

### Blood samples

At the initial visit - while fasting - venous blood samples will be collected using a McKesson Prevent® safety winged blood collection set (McKesson MFR# 2194) with BD Vacutainer® one-use holder (Becton Dickinson 64815), into 1x lithium heparin and 1x EDTA BD Vacutainer®. After identifying a suitable vein, and the site cleaned using an 70% isopropyl alcohol swab, a tourniquet will be applied briefly until venepuncture is achieved. Prolonged venous occlusion is to be avoided. All the following preparatory steps are to be completed within 5 minutes of the blood draw.

Analysis of frozen samples will be done as a batch after completion of recruitment.

#### RNA Transcriptomics

Within 2 minutes, using a Rainin Pipet-Lite L-1000 XLS pipette (Mettler Toledo), with 1000μl pipette tip (ART® BioProducts), an aliquot of 500μL of blood will be transferred from the EDTA vacutainer into a 5mL tube containing 2mL of a proprietary RNA Preservative Buffer (RBP, Viome Life Science, USA), capped and shaken vigorously for 30 seconds. After labelling, this is then stored at -20°C until transfer to a -80°C freezer.

With a new pipette tip, blood from the same EDTA tube is dripped to fill 4 circles on Whatman® protein saver cards (Sigma WHA10534612). After labelling and being allowed to dry at room temperature for 30 min, they are then placed in individual zip-lock bags with two 5g sachets of silica gel moisture absorbent (S-3905), sealed and stored at -20°C until transfer to a -80°C freezer.

#### Blood chemistry

From the lithium heparin vacutainer, 100μL of blood is transferred using Rainin Pipet-Lite L-300 XLS pipette (Mettler Toledo), fitted with a Rainin 300μL BioClean Ultra LTS pipette tip (Mettler Toledo), into the entry port on a fresh MetLac12 reagent disc (Abaxis®, USA, 400-1037) and immediately inserted into a Piccolo Xpress® analyser (Abaxis®) for basic blood chemistry (glucose, sodium, potassium, chloride, total carbon dioxide (CO_2_), calcium, phosphorus, magnesium, blood urea nitrogen, lactate, albumin, and creatinine).

The analyser is designed for point of care analysis of blood samples. Each single-use disc contains a diluent, which is automatically mixed with the blood sample, and using centrifugal and capillary forces into separated cuvettes, which contain the reagent beads specific for each analyte. Reaction products in the cuvettes are then measured photometrically. The equipment has inbuilt self-calibration and quality control functions, which are automatically done with each sample analysed.

#### Redox status

Finally, two separate blood samples (20µl and 50µl) will be collected from a capillary finger prick for evaluation of free oxygen radicals (FORT) and free oxygen radical defenses (FORD) according to the manufacturer’s instructions [27].

The FORT/FORD test is a reliable measure of whole blood oxidative stress [28]. It is a colorimetric assay, using disposables purchased as commercially available kits (BioMed Science/ Callegari Sr, Italy). The filled capillary tubes are immediately added to a 2mL tube with supplied reagents, centrifuged (CR6000), and analysed in the automated dedicated spectrophotometer (CR3000 RCH, FormPlus, Callegari, Parma, Italy).

FORT is an indirect measure of reactive by-products in whole blood. It is based on Fenton’s reaction, determining the capacity of transition metal ions (Fe^3+^ /Fe^2+^) to catalyse the breakdown of hydroperoxides (R-OOH) into derivative radicals [alkoxyl (R-O•) and peroxyl radicals (R-OO•)]. FORT units are expressed as equivalent concentrations of H_2_O_2_ mmol/L^-^1, with one FORT unit corresponding to approximately 7.6 mmol/L of H_2_O_2_ (equivalent to 0.26 mg/L) [27].

FORD is an indirect measure of the antioxidant capacity in whole blood. It is based on the capacity of ascorbic acid, glutathione, and albumin in the sample to reduce a preformed radical cation. FORD is expressed as mmol/l of Trolox (6-hydroxy-2,5,7,8-tetramethylchroman-2-carboxylic acid; a water-soluble analogue of vitamin E) [27].

### Stool

Participants will be asked to collect faecal samples as soon as practicable after the initial visit, while abstaining from probiotics and antibiotics. After first urinating, EasySampler® stool collector paper (GP Medical Devices, Holstebro, Denmark) is used to prepare a stool-catch platform. From the deposited stool, a pea-sized scoop will be taken with the provided scoop-spoon and immediately transferred to a pre-labelled stool tube (Sarstedt NC9574115, Fisher Scientific, Waltham, MA), containing 6 mL RNA preservative buffer (RPB) and 9-13 beads that facilitate mixing of the sample. After vigorously shaking the tube for 30 seconds, a 30-second rest period is followed by repeat shaking/resting two more times**.** This tube is then placed inside a white-capped stool tube (Sarstedt, Fisher Scientific NC1933356; Waltham, MA) containing an absorbent liner, then into a zip-lock bag and frozen at -20°C until transfer to -80°C freezer.

### Saliva

Saliva samples will be collected by spitting into a prelabelled sterile 5mL screw cap collection tube, (6101S, Growing Labs, Suwanee, GA) before cleaning teeth or eating in the morning. After collecting 1.5ml of liquid saliva, 1.5mL of RPB will be added, the sample shaken vigorously for 15 seconds, then stored in a zip-lock bag and frozen at -20°C until transfer to -80°C freezer.

### Environmental samples

To determine interactions with mould exposure in the home (and/or workplace), participants will be asked to take surface swabs from six unique sites in their indoor environment where mould is visible, or typically grows. A list of likely places will be provided. After collecting a sample with a sterile flocked swab (Hydraflock, 25-3206-H, Puritan Medical Products, Guildford, ME), the swab tip is snapped into a pre-labelled tube containing RPB. The site of sample collection is matched to the tube identifier on a logging sheet, including a description of the sample and site collected from.

### Metatranscriptomic analysis: blood, stool, saliva & environmental samples

Samples will be analysed by Viome Life Sciences using metatranscriptomics, developed for analysis of human clinical samples. The method has been described for faecal samples [29], saliva [30], and blood [31]. In this study, it will also be applied to environmental samples. After mixing with preservative (as detailed for each sample), the RNA is stable for up to 4 weeks at ambient temperature.

Prior to analysis, samples are lysed using a combination of mechanical (bead beating) and chemical lysis (detergents and denaturants). Most downstream methods are automated and performed by Hamilton Star liquid handlers. Total nucleic acids are purified from clarified lysates using a bead-based method. DNA is degraded enzymatically, and heat inactivated. Removal of non-informative RNAs (RNR) is done with a subtractive hybridisation method. Currently, 132 microbial probes that target microbial rRNAs and 80 human probes that target abundant human transcripts (rRNA, globin, etc.,) are utilised. Microbial probes are applied to stool and surface swab samples, human probes are applied to blood samples, and a combination of microbial and human probes are applied to saliva samples. After RNR, the remaining RNAs are converted into cDNA, then size-selected for Illumina sequencers using the spri bead technology. Final libraries are created with limited cycles of PCR that also adds unique dual barcodes to each sample. Libraries from individual samples are pooled and purified/size selected using either spri beads or Pippin prep (​​Sage Science). Library pools are sequenced on an Illumina NavSeq 6000 instrument using 2x150 sequencing chemistry. Bcl files are demultiplexed using bcl2fastq software and fastq files are processed using Viome Life Sciences proprietary, cloud-based software. The bioinformatic output from each sample type includes genome- and species-level taxonomic classification (including relative abundance), relative abundance of microbial Kyoto Encyclopaedia of Genes and Genomes (KEGG) orthologs, and quantitative expression of human genes.

Dried blood spot samples will be analysed using the Viome Multiplexed Immunoassay (VMI). This is a combination of serology and ELISA tests, using optically encoded Luminex beads for performing multiple analyses in one reaction. Currently, VMI comprises serology tests that quantify immunoglobulin (IgG) antibodies against 46 foods and human thyroid peroxidase, high sensitivity C-reactive protein (ELISA), and tumour necrosis factor-α (ELISA).

The VMI method starts with elution of whole blood from the dried blood spot (Whatman®) cards. The elution is incubated with beads for one hour, then the beads are washed. For serology tests, a detection antibody conjugated to phycoerythrin is added. For ELISA tests, the secondary antibody is added, followed by the detection antibody, also conjugated to phycoerythrin. Beads are counted and imaged using the MAGPIX system (Luminex) to obtain the median fluorescence intensity (MFI) for each test. Using calibration (standard) curves, the MFI values are converted to analyte concentrations.

### Urine samples

Intestinal hyperpermeability is a phenomenon in which leakage of molecules and ions below ~0.4nm [32] occurs through what are normally tight epithelial cell junctions in the gut lumen [33]. Increased paracellular leakage has been implicated in many disorders, including fibromyalgia [34].

At the initial appointment, a pack is provided with collection equipment, 3x 10mL Fisherbrand™ transport tube (Fisher Scientific 13-711-20, Globe Scientific, Mahwah, NJ) labelled #1, #2 & #3, and with identifier code. Detailed instructions, including a link to a video on YouTube and a checklist/log sheet is provided for recording of times and urine volumes.

The test involves a 4-hour urine collection, completed at home, with urine aliquots at baseline, and 2 and 4-hours after ingestion of a lactulose and mannitol challenge drink, as detailed by Sequiera et al [35]. Participants are instructed to avoid collecting while menstruating, abstain from non-steroidal anti-inflammatory drugs for at least two days, abstain from alcohol, aspirin, probiotics, and mannitol-containing foods (cauliflower, mushrooms, strawberries, onions, pumpkin, sweets, chewing gums or substances with artificial sweeteners) the day before testing and adhere to the collection protocol as outlined.

Other than water as detailed, no food or fluid is permitted until completion of sample collection. Participants will also be asked to note any symptoms occurring during the test and during the following 6 hours.

In a fasting state, from the first morning urination, a ~9mL sample is transferred into tube #1 and frozen as soon as possible.

The test substrates – 15mL lactulose BP 3.34g/5mL (Laevolac, Douglas Pharmaceuticals, NZ) and 5g d-mannitol AR (31501, ECP Labchem Ltd, NZ), dissolved in 250mL water - are then ingested. For the following 4 hours all urine passed is collected and transferred into a graduated 1L container (JC1000N, SprayPac, NZ) pre-spotted with 1mL chlorhexidine gluconate 20% (RN Laboratories, India; Amtrade NZ, FP/I/20/0895).

Two hours after taking the test solution, participants urinate and add all the urine passed to the 1L container provided, note the total volume, then extract 9mL, using a disposable graduated transfer pipette (Fisherbrand™, 13-711-20, Fisher Scientific, Waltham, MA). At this point they will drink 250mL of water and continue to collect all urine passed for a further two hours.

Four hours after ingesting the test substrate, participants will empty their bladder, adding all urine passed to the 1L container. After recording the total volume of urine now in the 1L container, a final 9mL sample will be pipetted off into the third tube. All samples will be stored frozen until returning them to the study site, where they are either centrifuged immediately or transferred to a -20°C freezer.

Thawed samples will be spun at 2075 relative centrifugal force for 5 minutes to remove debris and cellular particles. From each 9mL tube, urine will then be aliquoted into 2x 2mL microtubes (Scientific Specialties Inc., 3621-875, Interlab, Wellington, NZ) and baseline samples spotted with 2μL of 20% chlorhexidine. Labelled tubes are then transferred into cardboard freezer boxes before returning to the freezer.

For the analysis, recoveries of lactulose and mannitol will be determined using high performance liquid chromatography, after calibrating with specified concentrations of each. The full calibration procedure is outlined elsewhere [36]. The quantity of sugars in each urine sample is determined by multiplication of the relevant concentration (mg/mL) and the volume of accumulated urine at that time point. The result is expressed as a percentage of the original dose ingested.

### Breath testing

A study published in 2004 [37] demonstrated that 100% of participants with fibromyalgia had small intestinal bacterial overgrowth (SIBO) as determined by an oral lactulose challenge. To the best of our knowledge, this finding has not been replicated.

Fermentation of ingested carbohydrates by intestinal bacteria can result in gas production and may be associated with gastrointestinal distress [38]. We aim to conduct three separate hydrogen-methane breath tests on each participant. Initially, an oral glucose challenge, followed by fructose and lactulose challenges.

#### Breath test preparation

Prior to breath testing, four weeks must elapse after gastroenteritis, antibiotic therapy, or undergoing colonoscopy. Before commencing each test, participants will be asked to avoid probiotics and probiotic-containing foods for at least three days, follow a restricted low fermentable diet [39] for at least one full day, and fast overnight (12 hours minimum). On the day of each test participants will be asked to clean their teeth on awaking and avoid vigorous exercise. No food is permitted until sample collection is complete.

#### Conducting breath tests

All glucose breath tests will be conducted at the study site. These, and any subsequent breath tests conducted at the study site, will begin by collecting 3 baseline breath samples, at 1-minute intervals. To optimise gas exchange, each sample will be collected after a 15-second breath hold [40]. Participants expire into a GaSampler test kit (QuinTron, Milwaukee, WI [QT] 00892,) and 30cc of breath will be extracted from the sample holding bag with a leur-lock syringe (QT02741) with 1-way stopcock (QT01727-V). These end-alveolar samples are then injected into the BreathTracker SC (QuinTron) through a drying tube (QT01135-K) containing BreathPrep™ desiccant (QT01161-C), and a dust trap (QT01140-K).

At-home breath sample collection utilises the EasySampler test kit (QT04214) with detailed instructions and 13 sterile glass vials (QT02602). A single baseline sample precedes substrate ingestion.

For each breath test, whether at the study site or using an at-home kit, a single breath sample will be collected at 15-minute intervals after substrate ingestion, each after a 15-second breath hold. No fluid is permitted in the first 60 minutes after substrate ingestion; thereafter plain water may be taken during the first 10 minutes after collection of each breath sample. Food is not permitted until sample collection is complete.

Home-collected breath samples are to be returned to the study site within 5 days. This allows ample time for shipping delays, ensuring samples are received and analysed within the manufacturer’s specified 14-day viability period. Breath samples are extracted from the test tubes using the AlveoVac extraction system (QT02637), with vial adapter (QT02688) and dust trap (QT01140-K) fitted. Results are manually transcribed then entered into a Microsoft® Excel® spreadsheet.

The BreathTracker, maintained and calibrated in accordance with the manufacturer’s guideline [41], has a resolution of 1ppm for hydrogen (H_2_) and methane (CH_4_), and 2% for carbon dioxide (CO_2_). Accuracy is ± 3ppm or ± 5% of full scale for H_2_ and CH_4_, and ± 1% for CO_2_, linear range 0-150ppm for H_2_, 0-75ppm for CH_4_ and 0.1%-7% for CO_2_. The equipment automatically corrects samples for contamination with room or dead-space air by detecting the CO_2_ concentration, thus giving the true alveolar concentration of H_2_ and CH_4_ [41]. Where CO_2_ recoveries are insufficient, the sample is declared invalid. Data are reported in ppm, manually recorded, then entered into a Microsoft® Excel® spreadsheet for later analysis.

#### Symptoms during breath testing

For each breath test, a checklist will be completed to confirm participants’ adherence to pre-test instructions. Symptoms present at baseline are graded on a 0-10 scale, where 0 is “not present” and 10 is “the most severe”. The symptom record will be updated every 15 minutes, after collection of each breath sample.

#### Glucose breath test & oral glucose tolerance test

Glucose is a well-absorbed monosaccharide, used to detect bacterial overgrowth in the proximal small intestine [38]. An oral dose of 75g is used to confirm a diagnosis of diabetes mellitus (DM), and 50g is commonly administered during pregnancy to predict gestational DM [42].

For this test, 50g of anhydrous d-glucose (FCC-25304, ECP Labchem Ltd, NZ), dissolved with 250mL of water, is given after baseline data is collected, as per the most current guideline [38]. Breath samples are collected for 2.5 hours.

To monitor the glycaemic response, capillary samples will be obtained from a finger-prick at baseline, then 60, and 120 minutes after glucose ingestion with a single-use 23G disposable lancet (Unistik® 3, Owen Mumford, UK) and blood glucose monitor (CareSens® N, i-SENS, Inc., Seoul, Korea), range 1.1 - 33.3 mmol/L.

#### Fructose breath test

Fructose is a monosaccharide that is less readily absorbable than glucose. Early fermentation may indicate bacterial overgrowth in the proximal gut [43] and delayed fermentation suggests malabsorption, which may be associated with gastrointestinal distress [38]. For this test, 25g fructose (FCC-24704, ECP Labchem Ltd, NZ), dissolved in 250g of water, will be administered after capturing baseline data as above. Samples are collected for 3 hours.

#### Lactulose breath test

Lactulose is a synthetic disaccharide, for which humans lack an enzyme capable of digesting. Therefore, it passes into the colon unchanged, where it may act as an osmotic laxative and may also be fermented by colonic microbes. At doses that do not cause osmotic diarrhoea, lactulose has been used as a probe to evaluate oro-caecal transit time by onset of hydrogen production, with early rises in breath gases being considered predictive of SIBO [38]. The test substrate was 15mL of lactulose BP 3.34g/5mL (Laevolac, Douglas Pharmaceuticals, NZ) in 250mL of water. Sample collection is over 3 hours.

#### Survey data

Due to the complexity of fibromyalgia, and the multi-system symptoms that might affect a person with the condition, a comprehensive questionnaire has been developed on REDCap, which integrates the following validated tools:

- ACR fibromyalgia diagnostic criteria, 2016 [2]
- Revised Fibromyalgia Impact Questionnaire [44, 45]
- Rome IV [8, 46] – the current diagnostic criteria for FGIDs from the Rome Foundation
- Functional Bowel Disorder Severity Index [47, 48]
- Headache Symptom Questionnaire [49, 50]
- General Anxiety Disorder-2 [51]
- Medical Outcomes Study Sleep Scale [52]
- Short Form Survey -36 [53, 54] – for measures of health-related quality of life
- Oral Health Questionnaire [55]

Additional questions are included to capture other medical diagnoses, and symptoms related to environmental mould exposure. To assess current dietary intake, DietID®, a diet quality photo navigation tool will be used [56].

### Statistical analysis

All data will be anonymised and saved in password protected computer hosted on the University of Sydney’s Data Management System. Statistical analysis will be performed using specialist software. The level of significance will be set at *p* < 0.05.

Descriptive statistics will be used to detail the characteristics of the cohort, and the students’ t-test will be employed to compare participants and controls. Anthropometrics, symptom prevalence, scores and co-morbidities will be compared using univariate and multivariate analysis of variance, as appropriate.

After clustering data according to conventions appropriate to each of the questionnaires in the participant survey, group differences will be assessed using students’ t-tests, Wilcoxon signed-rank test chi-squared tests, and logistic regression, as appropriate.

For microbiome analyses, after stratification by age-groups to allow for variances that may be associated with reproductive life-stages, correction for multiple comparisons (e.g., using Benjamini–Hochberg FDR) and normalised operational taxonomy units will be evaluated using Pearson's correlations. Strength of associations with clinical variables will be tested using Spearman's Rho. Further statistical analysis of the data will be performed using machine learning methods.

## Discussion

This observational study will contribute extensive data on the interaction between the gut microbiota, digestive function, oxidative stress, diet, human gene expression and multiple symptoms associated with fibromyalgia in adult women. It will advance understanding of these inter-relationships and contribute to a deeper understanding of the aetiology of fibromyalgia and the role of the gut microbiome and its gene expression in this condition.

The study has the potential to shed light on areas where scientific understanding is currently lacking. It will contribute to a broader comprehension of the pathophysiology underlying fibromyalgia and its link to functional gut disorders. It may also contribute directly or indirectly to possible laboratory markers that can be used in either diagnosis or monitoring of fibromyalgia.

In addition, the findings will guide future intervention studies involving specific approaches, and potentially, microbiota-based therapies to improve outcomes for people living with fibromyalgia globally.

Limitations. Some level of attrition is expected, especially given the commitment required to complete the extensive testing outlined. Ideally, all samples collection should take place over 2-3 weeks; it is anticipated that not all participants will manage this. Any participant who does not complete the comprehensive study survey will be excluded. Any significant deviations from our study plan will be updated on the ANZCTR registry, with notification to HDEC if appropriate.

## Data Availability

Data sharing not applicable to this article as no datasets have yet been generated or analysed. Videos supporting participant collection of samples are hosted on YouTube: 1. Collection of urine samples for Lactulose-mannitol recovery test https://www.youtube.com/watch?v=THBWJgrfJmA 2. Conducting hydrogen-methane breath testing at home: https://www.youtube.com/watch?v=XZWR6Lf946Y

## Abbreviations

ACR: American College of Rheumatology
AHS: Australian Health Survey
ANZCTR: Australia and New Zealand Clinical Trials Registry
ATC/DDD: Anatomical Therapeutic Chemical/ Defined Daily Dose
BMI: Body mass index
cDNA: Complementary deoxyribonucleic acid
CH4: Methane
CO2: Carbon dioxide
DGBI: Disorders of Gut Brain Interaction
EDTA: Ethylenediamine tetraacetic acid
ELISA: Enzyme-linked immunosorbent assay
FGID: Functional gastrointestinal disorder
FIDGIT: Fibromyalgia, Digestive function and the microbiome of the Gastrointestinal Tract
FORD: Free oxygen radical defenses
FORT: Free oxygen radicals
H2: Hydrogen
H_2_O_2_: Hydrogen peroxide
HDEC: Health and Disability Committee
IBS: Irritable Bowel Syndrome
IgG: Immunogobulin G
KEGG: Kyoto Encyclopedia of Genes and Genomes
PCR: Polymerase chain reaction
RBP: RNA preservative buffer
REDCap: Research Electronic Data Capture
RNA: Ribonucleic acid
RNR: Removal of RNA
rRNA: Ribosomal RNA
SARS-CoV2: Severe acute respiratory syndrome coronavirus 2
SIBO: Small intestinal bacterial overgrowth
VMA: Viome Multiplexed Immunoassay

## References

[1] Inanici F, Yunus MB (2004). History of fibromyalgia: past to present. Curr Pain Headache Rep..

[2] Wolfe F, Clauw DJ, Fitzcharles M-A, Goldenberg DL, Häuser W, Katz RL (2016). 2016 Revisions to the 2010/2011 fibromyalgia diagnostic criteria. Semin Arthritis Rheum..

[3] Marques AP, Santo AdSdE, Berssaneti AA, Matsutani LA, Yuan SLK (2017). Prevalence of fibromyalgia: Literature review update. Rev Bras Reumatol Engl Ed..

[4] Skaer TL (2014). Fibromyalgia: disease synopsis, medication cost effectiveness and economic burden. PharmacoEconomics..

[5] Giorgi V, Sirotti S, Romano ME, Marotto D, Ablin JN, Salaffi F, et al. Fibromyalgia: one year in review. 2022. Clin Exp Rheumatol. 2022. 10.55563/clinexprheumatol/if9gk2. 10.55563/clinexprheumatol/if9gk2 35748720

[6] Goebel A, Andersson D, Barker C, Basu N, Bullock C, Bevan S (2022). Research recommendations following the discovery of pain sensitizing IgG autoantibodies in fibromyalgia syndrome. Pain Med..

[7] Bellato E, Marini E, Castoldi F, Barbasetti N, Mattei L, Bonasia DE (2012). Fibromyalgia syndrome: etiology, pathogenesis, diagnosis, and treatment. Pain Res Treat..

[8] Drossman DA, Hasler WL (2016). Rome IV; Functional GI disorders: Disorders of gut-brain interaction. Gastroenterology..

[9] Erdrich S, Hawrelak JA, Myers SP, Harnett JE. A systematic review of the association between fibromyalgia and functional gastrointestinal disorders. Therap Adv Gastroenterol. 2020;13. 10.1177/1756284820977402 10.1177/1756284820977402 PMC7727037 33343707

[10] Sperber AD, Atzmon Y, Neumann L, Weisberg I, Shalit Y, Abu-Shakrah M (1999). Fibromyalgia in the irritable bowel syndrome: Studies of prevalence and clinical implications. Am J Gastroenterol..

[11] Üçüncü MZ, ÇoruhAkyol B, Toprak D (2020). The early diagnosis of fibromyalgia in irritable bowel syndrome patients. Med Hypotheses..

[12] Mars RAT, Frith M, Kashyap PC (2021). Functional gastrointestinal disorders and the microbiome—What is the best strategy for moving microbiome-based therapies for functional gastrointestinal disorders into the clinic?. Gastroenterology..

[13] Minerbi A, Gonzalez E, Brereton NJB, Anjarkouchian A, Dewar K, Fitzcharles MA, Chevalier S, Shir Y. Altered microbiome composition in individuals with fibromyalgia. Pain. 2019;160(11):2589-602. 10.1097/j.pain.0000000000001640. 10.1097/j.pain.0000000000001640 31219947

[14] Erdrich S, Hawrelak JA, Myers SP, Harnett JE (2020). Determining the association between fibromyalgia, the gut microbiome and its biomarkers: A systematic review. BMC Musculoskelet Disord..

[15] Ticinesi A, Lauretani F, Milani C, Nouvenne A, Tana C, Del Rio D (2017). Aging gut microbiota at the cross-road between nutrition, physical frailty, and sarcopenia: Is there a gut–muscle axis?. Nutrients..

[16] Paray BA, Albeshr MF, Jan AT, Rather IA (2020). Leaky gut and autoimmunity: An intricate balance in individuals health and the diseased state. Int J Mol Sci..

[17] Schroeder BO, Bäckhed F (2016). Signals from the gut microbiota to distant organs in physiology and disease. Nat Med..

[18] Minerbi A, Gonzalez E, Brereton N, Fitzcharles MA, Chevalier S, Shir Y. Altered serum bile acid profile in fibromyalgia is associated with specific gut microbiome changes and symptom severity. Pain. 2023;164(2):e66-e76. 10.1097/j.pain.0000000000002694. 10.1097/j.pain.0000000000002694 35587528

[19] Ma N, Ma X (2019). Dietary amino acids and the gut-microbiome-immune axis: Physiological metabolism and therapeutic prospects. Compr Rev Food Sci Food Saf..

[20] Wu W-JH, Zegarra-Ruiz DF, Diehl GE. Intestinal microbes in autoimmune and inflammatory disease. Front Immunol. 2020;11. 10.3389/fimmu.2020.597966 10.3389/fimmu.2020.597966 PMC7786055 33424846

[21] Vatanen T, Kostic Aleksandar D, d’Hennezel E, Siljander H, Franzosa Eric A, Yassour M (2016). Variation in microbiome LPS immunogenicity contributes to autoimmunity in humans. Cell..

[22] Li B, Selmi C, Tang R, Gershwin ME, Ma X (2018). The microbiome and autoimmunity: a paradigm from the gut–liver axis. Cell Mol Immunol..

[23] Klemp P, Williams SM, Stansfield SA (2002). Fibromyalgia in Maori and European New Zealanders. Int J Rheum Dis..

[24] Almansa C, Rey E, Sanchez RG, Sanchez AA, Diaz-Rubio M (2009). Prevalence of functional gastrointestinal disorders in patients with fibromyalgia and the role of psychologic distress. Clin Gastroenterol Hepatol..

[25] WHO Collaborating Centre for Drug Statistics Methodology: ATC classification index with DDDs. WHO. 2021. Available from: https://www.whocc.no/atc_ddd_index/.

[26] Australian Food, Supplement and Nutrient Database (AUSNUT). FSANZ. 2011-13. Available from: https://www.foodstandards.govt.nz/science/monitoringnutrients/ausnut/ausnutdatafiles/Pages/dietarysupplements.aspx.

[27] Palmieri B, Sblendorio V (2010). Current status of measuring oxidative stress. Methods Mol Biol..

[28] Quinn KM, Cox AJ, Roberts LA, Briskey D, Minahan C (2021). Reliability of a point-of-care device to determine oxidative stress in whole blood before and after acute exercise: A practical approach for the applied sports sciences. J Sports Sci..

[29] Hatch A, Horne J, Toma R, Twibell BL, Somerville KM, Pelle B (2019). A robust metatranscriptomic technology for population-scale studies of diet, gut microbiome, and human health. Int J Genomics..

[30] Banavar G, Ogundijo O, Toma R, Rajagopal S, Lim YK, Tang K, et al. The salivary metatranscriptome as an accurate diagnostic indicator of oral cancer. NPJ Genomic Medicine. 2021;6(1). 10.1038/s41525-021-00257-x. 10.1038/s41525-021-00257-x PMC8654845 34880265

[31] Toma R, Duval N, Pelle B, Parks MM, Gopu V, Torres PJ (2020). A clinically validated human capillary blood transcriptome test for global systems biology studies. Biotechniques..

[32] Maxton DG, Bjarnason I, Reynolds AP, Catt SD, Peters TJ, Menzies IS (1986). Lactulose 51Cr-labelled ethylenediaminetetra-acetate, l-rhamnose and polyethyleneglycol 500 as probe markers for assessment in vivo of human intestinal permeability. Clin Sci (Lond)..

[33] Camilleri M (2019). Leaky gut: mechanisms, measurement and clinical implications in humans. Gut..

[34] Goebel A, Buhner S, Schedel R, Lochs H, Sprotte G (2008). Altered intestinal permeability in patients with primary fibromyalgia and in patients with complex regional pain syndrome. Rheumatology..

[35] Sequeira IR, Lentle RG, Kruger MC, Hurst RD (2014). Standardising the lactulose mannitol test of gut permeability to minimise error and promote comparability. PLOS ONE..

[36] Sequeira IR, Lentle RG, Kruger MC, Hurst RD (2012). The effect of aspirin and smoking on urinary excretion profiles of lactulose and mannitol in young women: toward a dynamic, aspirin augmented, test of gut mucosal permeability. Neurogastroenterol Motil..

[37] Pimentel M, Wallace D, Hallegua D, Chow E, Kong Y, Park S (2004). A link between irritable bowel syndrome and fibromyalgia may be related to findings on lactulose breath testing. Ann Rheum Dis..

[38] Hammer HF, Fox MR, Keller J, Salvatore S, Basilisco G, Hammer J (2022). European guideline on indications, performance, and clinical impact of hydrogen and methane breath tests in adult and pediatric patients: European Association for Gastroenterology, Endoscopy and Nutrition, European Society of Neurogastroenterology and Mot. United European Gastroenterol J..

[39] Erdrich S, Tan ECK, Hawrelak JA, Myers SP, Harnett J. Hydrogen-methane breath testing results influenced by oral hygiene. Sci Rep. 2020;11(1). 10.1038/s41598-020-79554-x. 10.1038/s41598-020-79554-x PMC7794545 33420116

[40] Strocchi A, Ellis C, Levitt MD (1991). Reproducibility of measurements of trace gas concentrations in expired air. Gastroenterology..

[41] QuinTron. BreathTracker SC operator manual. Milwaukee, WI: QuinTron Instrument Company, Inc.; 2019. p. 19.

[42] Phillips PJ (2012). Oral glucose tolerance testing. Aust J Gen Pract..

[43] Enko D, Kriegshäuser G, Kimbacher C, Stolba R, Mangge H, Halwachs-Baumann G (2015). Carbohydrate malabsorption and putative carbohydrate-specific small intestinal bacterial overgrowth: Prevalence and diagnostic overlap observed in an Austrian outpatient center. Digestion..

[44] Bennett RM, Friend R, Jones KD, Ward R, Han BK, Ross RL (2009). The Revised Fibromyalgia Impact Questionnaire (FIQR): validation and psychometric properties. Arthritis Res Ther..

[45] Burckhardt CS, Clark SR, Bennett RM (1991). The fibromyalgia impact questionnaire: development and validation. J Rheumatol..

[46] Palsson OS, Whitehead WE, van Tilburg MAL, Chang L, Chey W, Crowell MD (2016). Development and validation of the Rome IV diagnostic questionnaire for adults. Gastroenterology..

[47] Drossman DA, Li Z, Toner BB, Diamant NE, Creed FH, Thompson D (1995). Functional bowel disorders. A multicenter comparison of health status and development of illness severity index. Dig Dis Sci..

[48] Sperber AD, Carmel S, Atzmon Y, Weisberg I, Shalit Y, Neumann L (2000). Use of the Functional Bowel Disorder Severity Index (FBDSI) in a study of patients with the irritable bowel syndrome and fibromyalgia. Am J Gastroenterol..

[49] van der Meer HA, Visscher CM, Vredeveld T, Speksnijder CM, Nijhuis van der Sanden MW, Hh Engelbert R (2019). The diagnostic accuracy of headache measurement instruments: A systematic review and meta-analysis focusing on headaches associated with musculoskeletal symptoms. Cephalalgia..

[50] Headache Classification Committee of the International Headache Society (IHS). (2018). The International Classification of Headache Disorders, 3^rd^ edition. Cephalalgia..

[51] Plummer F, Manea L, Trepel D, McMillan D (2016). Screening for anxiety disorders with the GAD-7 and GAD-2: a systematic review and diagnostic metaanalysis. Gen Hosp Psychiatry..

[52] Hays RD, Stewart AL. Sleep measures. In: Stewart AL, Ware JEJ, editors. Measuring functioning and well-being: The medical outcomes study approach. Duke University Press: Duke University Press; 1992. p. 235–59. https://doi.org/10.7249/CB361.

[53] McHorney CA, Ware JE, Lu JFR, Sherbourne CD (1994). The MOS 36-Item Short-Form Health Survey (SF-36): III. Tests of data quality, scaling assumptions, and reliability across diverse patient groups. Med Care..

[54] Hays RD, Sherbourne CD, Mazel R. User's manual for the medical outcomes study (MOS) core measures of health-related quality of life. Santa Monica, CA. : RAND Corporation; 1995. Available from https://www.rand.org/pubs/monograph_reports/MR162.html

[55] Petersen PE, Baez RJ, World Health Organization. Oral health surveys: Basic methods. 5^th^ ed. Geneva: World Health Organization; 2013. Available from https://apps.who.int/iris/bitstream/handle/10665/97035/97

[56] Bernstein A, Moore R, Rhee L, Aronson D, Katz D (2021). A digital dietary assessment tool may help identify malnutrition and nutritional deficiencies in hospitalized patients. Res Ideas Outcomes..

